# A systems genetics study of swine illustrates mechanisms underlying human phenotypic traits

**DOI:** 10.1186/s12864-015-1240-y

**Published:** 2015-02-14

**Authors:** Jun Zhu, Congying Chen, Bin Yang, Yuanmei Guo, Huashui Ai, Jun Ren, Zhiyu Peng, Zhidong Tu, Xia Yang, Qingying Meng, Stephen Friend, Lusheng Huang

**Affiliations:** Jiangxi Agricultural University, Nanchang, Jiangxi China; Genetics and Genomic Sciences, Icahn Institute for Genomics and Multiscale Biology, Icahn School of Medicine at Mount Sinai, New York, NY 10029 USA; BGI, Shenzhen, Guangdong China; Department of Integrative Biology and Physiology, University of California at Los Angeles, Los Angeles, CA USA; Sage Bionetworks, Seattle, WA USA

**Keywords:** Systems genetics, Swine model, Complex human traits

## Abstract

**Background:**

The pig, which shares greater similarities with human than with mouse, is important for agriculture and for studying human diseases. However, similarities in the genetic architecture and molecular regulations underlying phenotypic variations in humans and swine have not been systematically assessed.

**Results:**

We systematically surveyed ~500 F2 pigs genetically and phenotypically. By comparing candidates for anemia traits identified in swine genome-wide SNP association and human genome-wide association studies (GWAS), we showed that both sets of candidates are related to the biological process “cellular lipid metabolism” in liver. Human height is a complex heritable trait; by integrating genome-wide SNP data and human adipose Bayesian causal network, which closely represents bone transcriptional regulations, we identified *PLAG1* as a causal gene for limb bone length. This finding is consistent with GWAS findings for human height and supports the common genetic architecture between swine and humans. By leveraging a human protein-protein interaction network, we identified two putative candidate causal genes *TGFB3* and *DAB2IP* and the known regulators *MESP1* and *MESP2* as responsible for the variation in rib number and identified the potential underlying molecular mechanisms. In mice, knockout of *Tgfb3* and *Tgfb2* together decreases rib number.

**Conclusion:**

Our findings show that integrative network analyses reveal causal regulators underlying the genetic association of complex traits in swine and that these causal regulators have similar effects in humans. Thus, swine are a potentially good animal model for studying some complex human traits that are not under intense selection.

**Electronic supplementary material:**

The online version of this article (doi:10.1186/s12864-015-1240-y) contains supplementary material, which is available to authorized users.

## Background

The pig is one of the most important agriculture animals, which has provided the largest amount of consumable red meat protein. The pig is also a valuable model for studying human diseases because pigs are more similar in genomic structure to human than mice are [1]. Pigs are used as models in genetics analysis and gene knockout or knock-in studies of human diseases such as cystic fibrosis, Alzheimer’s disease, and *BRCA1*-associated mammary carcinogenesis. Genetic studies of pig models of human diseases led to the identification of novel quantitative trait loci for cutaneous melanoma and a novel mutation in the LDL receptor that contributes to spontaneous hypercholesterolemia [2]. Gene expression profiling in pig models led to the identification of *RACK1* as a potential marker of malignancy for human melanocytic proliferation [3]. The pig has also been used to study autoimmune, congenital, and bone diseases, as well as cancer, diabetes, and cardiovascular diseases such as atherosclerosis and hypertension.

However, the genetic similarity between pig and human has not been assessed in large, systematic studies. Thus, pigs may be underused as models for human diseases. Here we surveyed about 500 F2 animals in a swine cross using a high density 60 K SNP array and examined phenotypic traits of interests to both agriculture and human diseases, including anemia traits, limb bone length, and number of ribs.

Integrated network or systems biology approaches, which combine genetic, genomic, and phenotypic data into network views, have been applied to understand obesity [4,5], cancers [6], and other human diseases. Integrated network approaches are powerful tools for analyzing complex high-throughput data. They have provided many new insights into diseases [7] and identified many novel candidate genes that cause human diseases [8], and were later validated experimentally [9].

By applying integrated analysis on phenotypic traits and genotype data, we show that both swine and human genome wide association candidates for anemia traits are related to lipid metabolism in liver. By integrating phenotype and genotype data with human adipose Bayesian causal network, we identified *PLAG1* for limb bone length (corresponding to human height). We then integrated genetic association result and human protein-protein interaction network to identify two novel candidate causal genes *TGFB3* and *DAB2IP* as well as the known regulators *MESP1* and *MESP2* as responsible for the variation in rib number and illustrated the potential underlying molecular mechanisms. *Tgfb3* knockout together with *Tgfb2* in mice decreases number of ribs, which supports *TGFB3* as a regulator for rib number in pig.

## Results

A large-scale F2 intercross comprising 1,912 pigs was constructed by crossing the western breed White Duroc and the Chinese breed Erhualian [10]. These breeds differ in growth, fat, meat quality, and other phenotypic traits. Phenotypic traits including anemia traits, number of ribs and limb bone length were measured at day 240 (Methods).

High density SNP genotypes for 497 F2 animals were successfully generated. Of 52,183 SNPs (52,077 SNPs on the 60 K chip [11] and 106 internally developed SNPs), 11,718 informative SNPs were selected for further analysis based on their call rates, minor allele frequencies, and Hardy-Weinberg equilibrium (HWE) tests (Methods). The average distance between informative SNP markers was 181 kb (median 105.6 kb). We therefore defined the most likely regions of major loci as regions within 200 kb on each site of the most significant SNPs in genome-wide association results. To scan phenotypes against the genome-wide SNP genotype data for association between trait and SNP, we used the single-marker mixed model [12] (Methods). At a false discovery rate (FDR) <5% (corresponding *P* = 4.85 × 10^−6^), there were 12, 5, and 2 quantitative trait loci (QTLs) for anemia, bone length, and rib number traits, respectively. Then, we applied systems biology approach to identify potential causal genes underlying QTLs of phenotype traits.

### Causal genes for anemia traits

**Figure 1:**
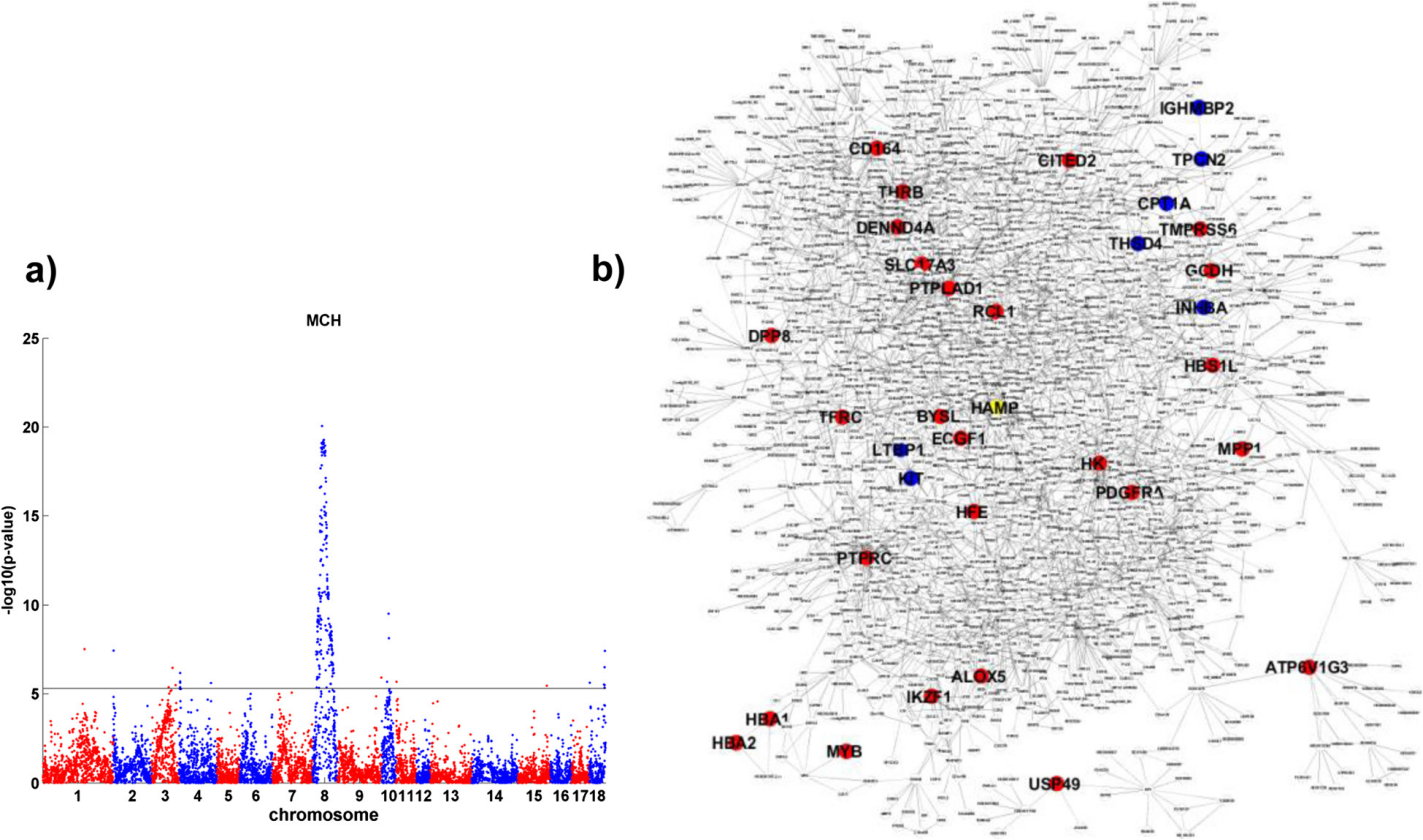
**Comparison of pig and human genetic architecture of anemia related traits such as mean corpuscular hemoglobin (MCH). a)** Pig SNP association result for MCH. There is a strong QTL at chromosome 8 along with 11 other significant QTLs. The black line represents a p-value threshold 4.85 × 10^−6^, corresponding to FDR = 0.05. **b)**. Both pig genome wide association candidates (blue nodes) and human GWAS candidate (red nodes) for MCH are in a liver subnetwork that involves in lipid metabolism. Hepcidin (*HAMP*, yellow node) is a sensor for iron and inflammation. The subnetwork provides a molecular link between anemia and lipid metabolism.

Hematopoietic disorders are associated with a variety of human diseases such as coronary heart disease, diabetes, and liver diseases. One founder breed of our F2 resource population, the White Duroc pig, has the dominant coat color and lower hemoglobin concentration, an indicator of macrocytic anemia [13]. Two blood parameters related to macrocytic anemia, mean corpuscular volume (MCV) and mean corpuscular hemoglobin (MCH) at day 240, were recorded for this F2 cross. Since MCV and MCH are tightly correlated (correlation coefficient = 0.89, *P* = 1.76 × 10^−169^), we used MCH as the representative of anemia traits in the downstream analysis. We previously identified a significant QTL for MCH at day 240 in a 3-cM region on SSC8 using 194 microsatellite markers [14]. In the current study, the SNP association results of MCH revealed 12 significant loci (Additional file 1: Table S1); the strongest association was on SSC8 (Figure 1a), as in our previous results. The QTL for MCH peaked at SNP marker MARC0034580 (SSC8:43.43 Mb). The 200 kb flanking region on each side of the marker contains only one gene, *KIT*, a finding which suggests *KIT* is the causal gene for MCH at locus SSC8:43.43 Mb. *KIT* regulatory mutations, including a gene duplication and a splice mutation that leads to the skipping of exon 17, are responsible for the dominant white phenotype in pigs [15]. These regulatory mutations have profound pleiotropic effects on peripheral blood cell measures in Western commercial pigs [13]. The second strongest association was centered at MARC0090810 (SSC10:38.4 Mb). The 200 kb flanking region on each side contains only one gene, *ACO1*, which encodes aconitase 1. Also known as iron regulatory element binding protein 1 (*IREB1*), *ACO1* regulates cellular iron homeostasis and is linked to anemia in human [16].

In addition, there were multiple significant QTLs for MCH, suggesting complex genetic regulation of the MCH trait. Multiple loci for MCH have been identified in human GWAS [17-19] (Methods, Additional file 2: Table S2). However, only one gene, *KIT*, was present in the candidate sets from human GWAS and from our swine cross. Bone marrow, kidney, and liver are important tissues for red blood cell production and homeostasis. Instead of directly comparing the two candidate sets from swine and human, we examined how these genes are regulated in a human liver transcriptional network (see Methods for details). 7 swine and 28 human GWAS candidates for MCH were included in the human liver network. The average shortest distances were 4.95 and 5.85, corresponding to empirical p-values = 0.01 and 0.067 (Methods) for the swine and human GWAS candidates, respectively, suggesting the swine candidate genes were likely to be transcriptionally co-regulated in human liver. We then sought to determine whether candidate genes identified in swine and human GWAS involve in similar subnetworks and similar biological processes. Subnetworks around swine candidates for MCH were significantly enriched in the Gene Ontology (GO) biological process cellular lipid metabolism (fold enrichment =3.5, Fisher’s Exact Test p-value= 1.2 × 10^−11^, and EASE score [20] = 4.5 × 10^−11^, detailed in Supplementary Results) while subnetworks around human GWAS candidates were enriched in the GO biological processes immune response and lipid metabolism (fold enrichments = 1.78 and 1.55, Fisher’s Exact Test p-values= 3.3 × 10^−16^ and 1.1 × 10^−6^, EASE scores = 7.8 × 10^−16^ and 1.8 × 10^−6^, respectively) . The enrichment of multiple GO biological processes in the human GWAS candidates subnetwork explains why human GWAS candidates were not significantly co-regulated in the human liver network as a whole (empirical p-value = 0.067 as shown above). The swine candidate subnetwork and human candidate subnetwork overlapped significantly (fold enrichment = 2.0, Fisher’s Exact Test p-value = 6.3 × 10^−9^, EASE score= 1.3 × 10^−8^), and genes in the GO biological process cellular lipid metabolism were even more enriched when considering the two subnetworks together (fold enrichment = 1.8, Fisher’s Exact Test p-value = 6.3 × 10^−12^, EASE score = 1.3 × 10^−11^) (Figure 1b). Anemia has been linked to lipid profiles such as cholesterol and apolipoprotein levels, triglycerides, and lipid peroxidation in both animal and human studies [21-28]. Many genes in the anemia-associated subnetwork we identified participate in diverse lipid-related functions, such as cholesterol biosynthesis, lipid transport, and lipid oxidation, providing mechanistic support for the phenotypic connections between anemia and lipid metabolism. In addition, anemia, inflammation, and obesity have been linked in many studies [29] and are linked transcriptionally [4,5]. Hepcidin (encoded by *HAMP*, yellow node in Figure 1b) is a body sensor for iron and inflammation [30] and is increased in obese individuals [31], which is a potential molecular connection between anemia and inflammation.

In sum, our results suggest that both swine and human candidate genes for the anemia trait MCH involve in a similar subnetwork related to lipid metabolism, which supports a link between anemia and obesity.

### Identification of *PLAG1* and *HMGA1* as causal genes for pig limb bone length and human height

Human height is a typical polygenic trait. Hundreds of loci that affect human height have been identified in GWAS [32]. However, there are no good models for studying mechanisms by which these loci affect human height. We illustrated here that QTLs for the length of pig limb bones are in good concordance with human GWAS results, suggesting that the pig is such a model.

**Figure 2:**
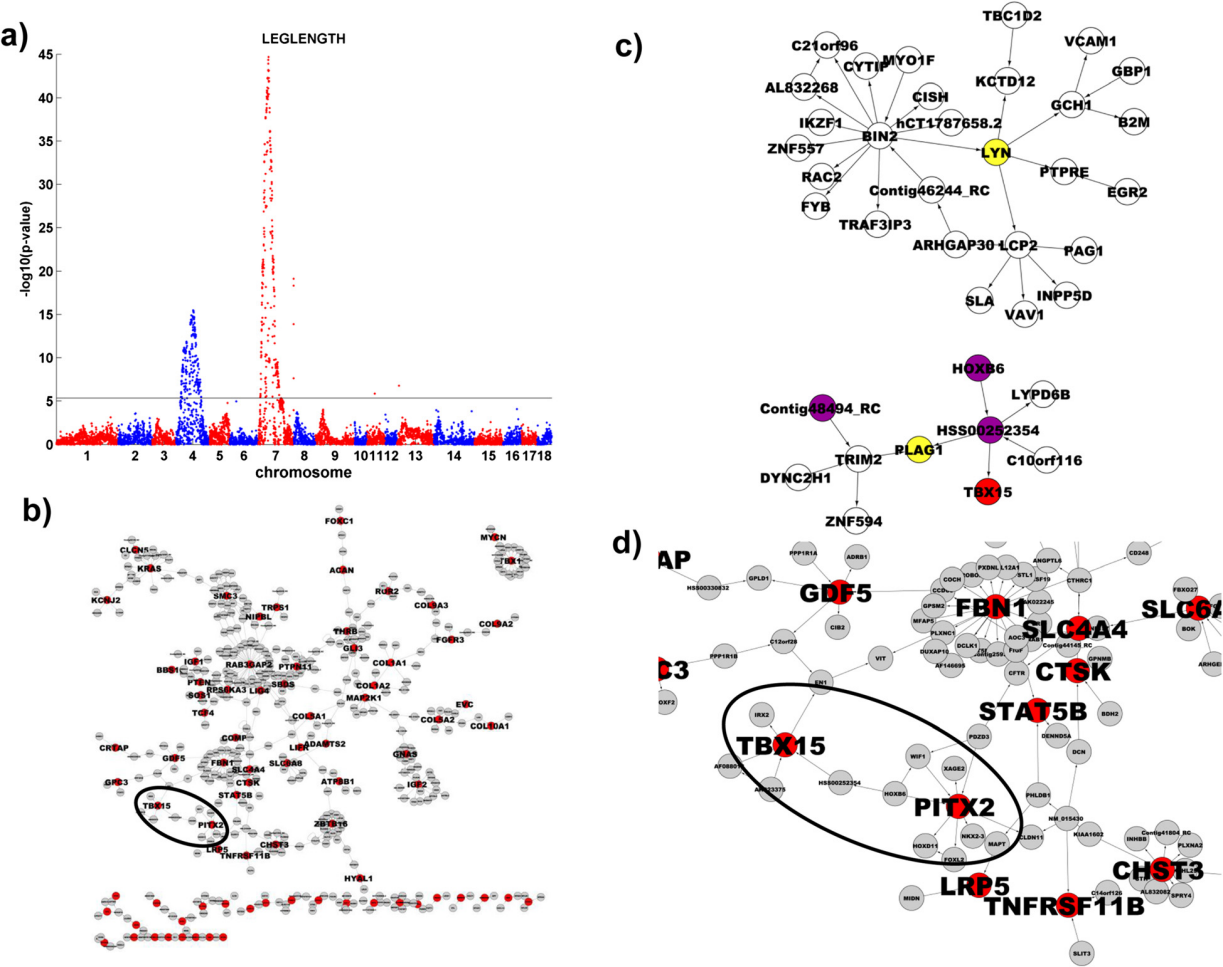
**Genome-wide association result for the limb bone length. a)** Global view of the association result shows that two major loci on chromosomes 4 and 7 affect the limb bone length. **b)** Subnetworks of height-related genes based on OMIM in the human adipose transcriptional network. **c)** Subnetworks of genes at the chromosome 4 locus in the human adipose network. **d)** Zoom-in view of the region in the height-related OMIM gene subnetwork that overlaps with the *PLAG1* subnetwork. Red nodes are height-related OMIM genes. Yellow nodes in 2c are genes mapped to the chromosome 4 locus. Purple nodes are nodes in the OMIM gene subnetwork in 2b.

Two loci were strongly associated with limb bone length (Figure 2a, Additional file 3: Table S3). These loci were centered at SSC4:82.65 Mb and SSC7:35.18 Mb with p-values of 3.37 × 10^−16^ and 2.07 × 10^−45^, respectively, consistent with the result of a previous QTL study on the length of individual bones [33]. The flanking regions (200 kb on each side) of chromosomes 4 and 7 loci contain 7 genes (*SDR16C5*, *RPS20*, *PLAG1*, *PENK*, *MOS*, *LYN*, and *CHCHD7*) and 5 genes (*SPDEF*, *RPS10*, *PACSIN1*, *HMGA1*, and *C6orf106*), respectively. Leg length is generally proportional to height. Our results are similar to those of GWAS of human height. The syntenic regions on human genome of the two loci we identified in pigs matched perfectly with two human loci (8q12 and 6p21) associated with human height in GWAS [32,34]. The concordant results of genetic studies of pig and human indicate that these loci have profound effects on bone development, so it is worth identifying causal candidate genes at these loci. *HMGA1* (at SSC7:35.18 Mb for pig and 6p21 for human) has been suggested as the causal gene for height, possibly through a mechanism involving modification of chromatin structure [32]. However, it is unclear which gene or genes at locus 8q12 are causal for human height [32,35]. Therefore, we compared subnetworks around swine candidate genes with the subnetwork derived from genes known to affect human height.

We selected 241 genes potentially related to human height on the basis of their disease associations in the OMIM database (http://www.ncbi.nlm.nih.gov/Omim). Osteoblasts and adipocytes are very close in cell lineage [36] and can be converted to each other by molecular signals [37]. Subnetworks in a human gene regulatory network for omental fat representing a bone regulatory network (Methods) were extracted by using the 241 OMIM genes as seeds (Figure 2b). 82 of the 241 genes were included in the omental fat network. The largest subnetwork in Figure 2b contained 50 of the 82 OMIM genes, which indicates that height related genes are coherently regulated in ometal fat. Using genes at the human 8q12 locus as seeds, we extracted subnetworks from the omental fat network as well (Figure 2c). There were two subnetworks, one centered on *LYN* and one on *PLAG1*. Only *PLAG1* subnetwork overlapped with the height-related OMIM gene subnetwork: 4 of 10 nodes were in the OMIM subnetwork (fold enrichment = 7.8, Fisher’s Exact Test p-value = 7.3 × 10^−4^, EASE score = 0.0063; the zoom-in view of the overlap between *PLAG1* subnetwork and the OMIM height gene subnetwork is shown in Figure 2d). This result strongly suggests that *PLAG1* is a candidate gene at the 8q12 locus. *Plag1* knockout mice have reduced litter weight and retarded embryonic and postnatal growth [38], which further implicates *Plag1* in regulating body size. In addition, variants modulating the expression of a chromosome domain encompassing *PLAG1* influence stature in cattle [39]. Some studies suggest that *PLAG1* is associated with human height [35], while others suggest *SDR16C5* as the causal gene at the 8q12 locus [32]. Our network analysis result objectively indicates that *PLAG1* is a causal gene for human height.

### Identification of *TGFB3*, *DAP2IP*, *MESP1*, and *MESP2* as causal genes for the number of ribs

**Figure 3:**
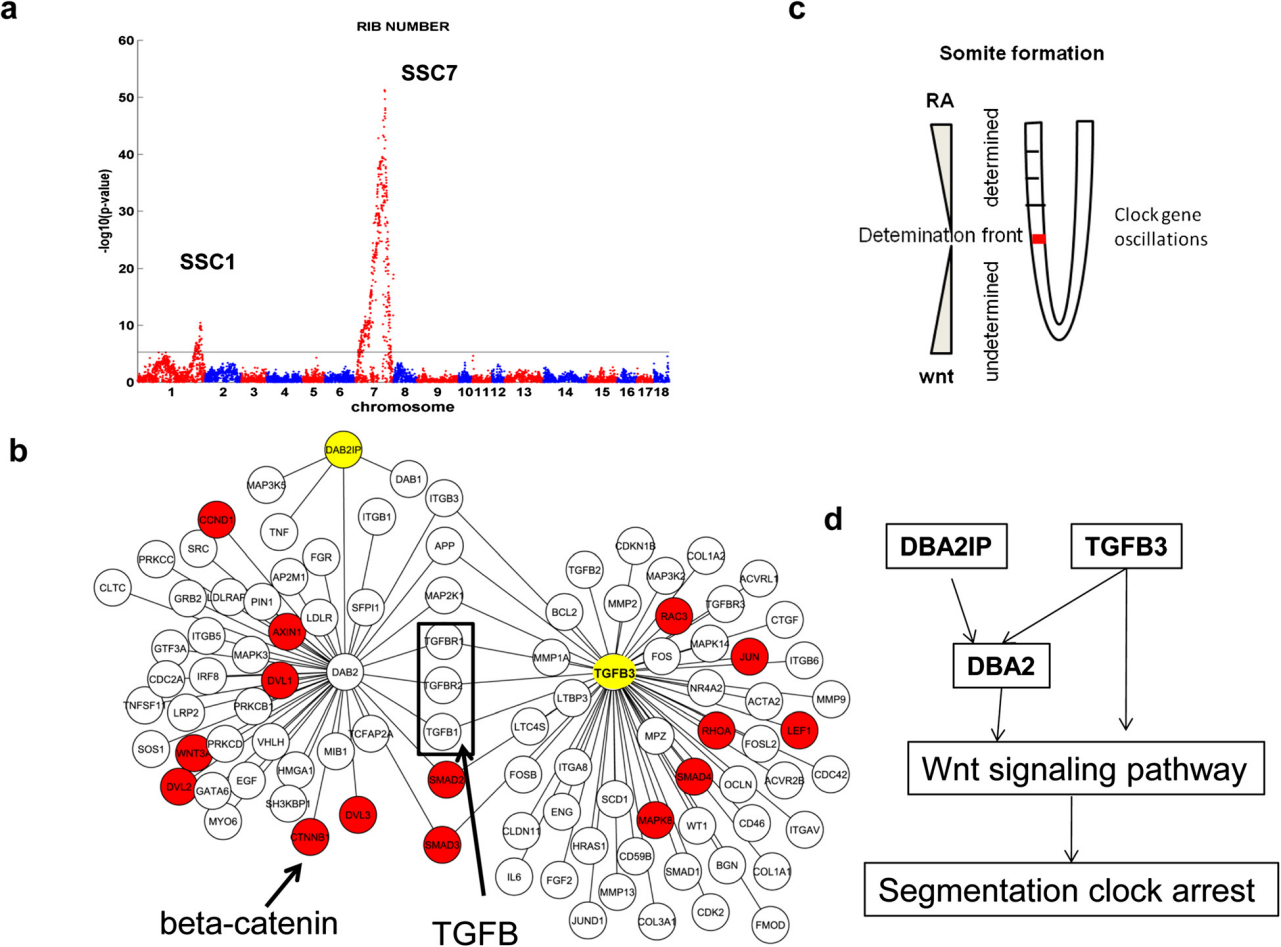
**Schematic diagram of the mechanism for regulating rib number. a)** The genome-wide association result shows that there are two significant loci on chromosomes 1 and 7 for rib number. **b)** The protein-protein interaction network around genes mapped to the chromosomes 1 and 7 loci (yellow nodes). The DAB2-DAB2IP and TGFB3 subnetworks overlap and are enriched for genes (red nodes) in the Wnt signaling pathway (fold enrichment = 20, Fisher’s Exact Test p-value= 9.99 × 10^−16^, EASE score = 2.1 × 10^−14^). **c)** Schematic graph showing somite formation (adapted from [47]). Vertebrae form during somitogenesis. The Wnt signaling pathway is critical for maintaining and stopping clock oscillation. **d)** Hypothetically, the chromosome 1 and 7 loci affect the number of ribs through an interaction between DAB2IP-DAB2-TGFB3 and the Wnt signaling pathway.

Pigs have 13 to 16 ribs [40], and meat production increases with extra ribs [40]. In human, one extra rib can increase cancer risk by 120-fold [41]. Previous studies indicate that loci on chromosomes 7 and 11 affect the number of ribs in pigs [42-44]. The number of vertebrae and the number of ribs are tightly correlated. Two major QTLs for the number of vertebrae were found on chromosomes 1 (SSC1:293.4 Mb) and 7 (SSC7:105.4 Mb), and *NR6A1* and *VRTN* were suggested as the causal genes underlying the two loci, respectively [45,46]. In our F2 intercross, we recorded rib number, and tested its association with the SNP genotypes. Our SNP association results (Figure 3a, Additional file 4: Table S4) also revealed two loci on chromosomes 1 and 7.

The chromosome 7 locus had the strongest association with rib number (*P*= 4.7 × 10^−52^) and is centered on marker ALGA0044022 at 105.38 Mb (Additional file 5: Figures S1a and b). Genes within the flanking region (200 kb up- and downstream) of the SNP marker are *TGFB3, IFT43* (*C14orf179*), and *C14orf118*. The chromosome 1 locus (association *P*= 3.56 × 10^−11^) is centered on the marker DRGA0002465 at 293.93 Mb. The flanking region (200 kb on each side of the marker) contains one gene, *DAB2IP*.

To prioritize the candidate genes at these loci, we explored the network structures around them. Transcriptional regulatory networks of mature tissues may not reflect regulation during early embryo development. However, protein-protein interactions (PPI) are not identified in a specific physiological state and thus may capture interactions or regulations during embryonic development. We therefore collected PPIs from multiple sources (Methods). The PPI subnetwork around the candidate genes (shown in Figure 3b) was significantly enriched for genes in the KEGG Wnt signaling pathway (fold enrichment = 20, Fisher’s Exact Test p-value= 9.99 × 10^−16^, EASE score = 2.1 × 10^−14^). In the early development of vertebrate embryos, the thoracic spine forms during somitogenesis, a process controlled by segmentation clock, whose key regulators include Notch, Wnt, and FGF [47] (Figure 3c). We found that *TGFB3* and *DAB2IP* interact with genes in the Wnt signaling pathway in the PPI subnetwork (Figure 3b), suggesting that these genes are the candidate genes for the two loci.

*Tgfb2* and *Tgfb3* overlap in function and compensate for each other. In *Tgfb2* knockout mice, *Tgfb3* regulates rib number. *Tgfb3*^*+/−*^ and *Tgfb3*^*−/−*^ mice have fewer ribs than their wildtype littermates [48]. *TGFB3* plays a key role in embryogenesis, and abnormalities in this and other genes in the FGF pathway contribute to human diseases such as oral cleft [49]. DAB2IP (disabled homolog 2 interacting protein) interacts with DAB2 [50]. *DAB2* plays an essential role in mesoderm differentiation [51] and inhibits Wnt/beta-catenin signaling in embryos [52]. A beta-catenin (CTNNB1) gradient determines the arrest of clock oscillation and maturation of somitogenesis [53]. Besides affecting the common Wnt signaling pathway, *TGFB* regulates the translation of *DAB2* mRNA [54]. We hypothesized that both loci influence rib number by interacting with the Wnt signaling pathway and then affecting the beta-catenin gradient and maturation of somitogenesis (Figure 3c and d).

**Figure 4:**
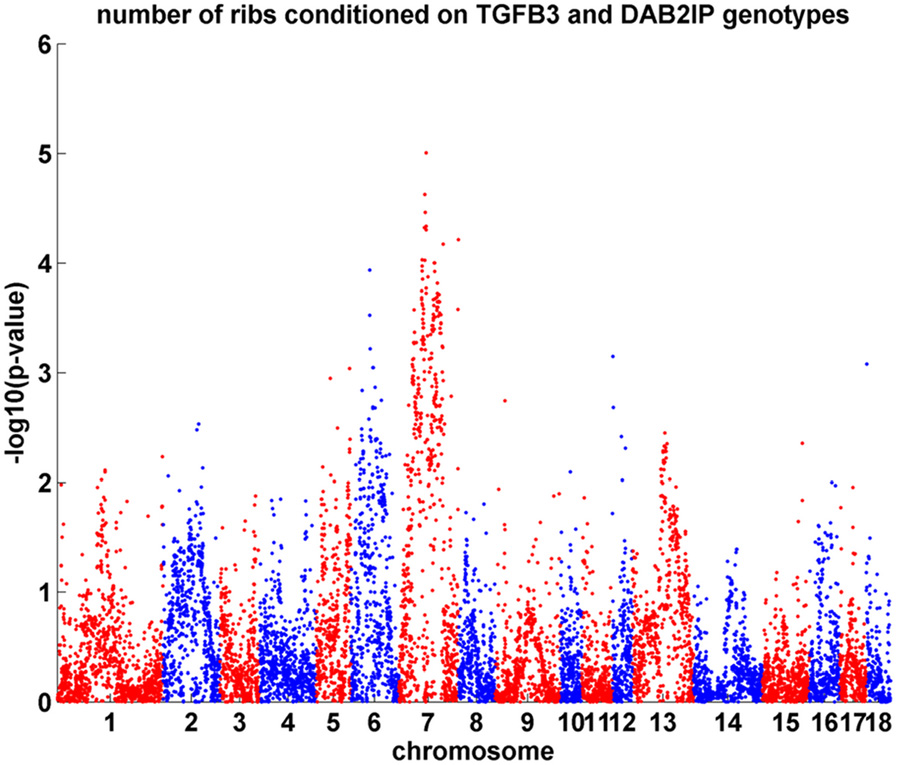
**Genome-wide association result for rib number conditioning on the genotypes at** ***TGFB3*** **and** ***DAB2IP*** **loci.** There is a significant locus at SSC7:60.8 Mb (p-value= 9.8 × 10^−6^). The p-value for the interaction between this locus (SSC7:60.8 Mb) and the TGFB3 locus (SSC7:107.3 Mb) is 0.02.

Somitogenesis is regulated by the Notch, Wnt and FGF pathways [47]. The mechanisms of both potential regulators, *TGFB3* and *DAB2IP*, are related to the Wnt and FGF pathways but not to the Notch pathway. To further explore causal regulators for the number of ribs, we regressed the number of ribs on the genotypes at *TGFB3* and *DAB2IP* loci using the following model *y* = *μ* + *sex* + *batch* + *G* + *g*_*TGFB*3_ + *g*_*DAB*2*IP*_ + *e*, where *y* is the number of ribs, *μ* is the mean, *G* is the kinship, *g*_*TGFB*3_ and *g*_*DAB*2*IP*_ are the genotypes at the *TGFB3* and *DAB2IP* loci, and *e* is the residual. We then tested whether other loci and their interactions with *TGFB3* and *DAB2IP* loci explain the residual variance *e* using the following models *e* ~ *g* + *g* * *g*_*TGFB*3_ and *e* ~ *g* + *g* * *g*_*DAB*2*IP*_. At SNP marker DBKK0000285 (SSC7: 60.2 Mb), there was one locus whose genotype and interaction with the *TGFB3* locus was associated with the residual variance (*P*= 9.8 × 10^−6^) (Figure 4). The flanking region of this locus contains eight genes: *WDR93*, *PLIN1*, *PEX11A*, *MESP2*, *MESP1*, *C7H15ORF38*, *APN*, and *AP2S2*. Among them, *MESP1* and *MESP2* are known regulators in somitogenesis [55], during which *MESP2* is a key regulator of the Notch pathway [56]. Thus, *MESP1* and *MESP2* are likely to be causal genes at this locus for rib number in pigs and they interact with the *TGFB3* locus to regulate both the Notch and Wnt signaling pathways.

## Discussion

This study shows that causal genes for traits of interests to both agriculture and human diseases can be identified by combining high-density SNP genotyping and Systems Biology approaches. By comparing pig and human candidate genes from GWAS for phenotypic traits related to anemia, bone length (equivalent to human height), and rib number, we identified putative causal genes and uncovered the mechanisms for these phenotypes. Specifically, we showed that both human and pig causal genes for anemia are related to lipid metabolism in liver. We confirmed that *HMGA1* and *PLAG1* are candidate genes for bone length, which corresponds to human height.

In genomic structure [1] and physiologically, pigs are more similar to humans than are mice, one of the most widely used models for human pathophysiology studies. Pig organs are similar to their human counterparts in size and shape and are a potential source for human organ transplants. Humans and pigs also have similar blood lipid profiles, whereas mice lack high density lipoprotein (HDL) particles. A study based on a complete pig genome assembly [57] shows that selection pressure in pigs is closer to that in humans than in mice. The dN/dS ratio (the ratio of the rate of non-synonymous substitutes to the rate of synonymous substitutions) is 0.144, 0.163, and 0.116 for the pig, human, and mouse, respectively [57]. These lines of evidence suggest that the pig is a good model for studying complex human diseases. Indeed, we found that genetic complexity of human disease related traits in the pig is similar to the complexity in human. For example, 22 loci are significantly associated with MCH/MCV in human GWAS [17-19]. We found 12 loci associated with MCH at genome-wide significance in the pig. In contrast, only one or two loci are associated with MCH in mouse crosses [58,59]. On the other hand, the mouse is still an important model for biomedical research and has unique advantages, including a shorter life and hence experimental cycle, greater availability of genetic, genomic, and molecular resources, and ease of genetic manipulation. The mouse is also a better model for target validation. Thousands of knockout or transgenic mice have been generated, and can be used to assess physiological and molecular changes due to genetic perturbations.

Even though there are 112 naturally occurring disease-causing mutations common to humans and pigs [57], a large overlap between most of disease causal loci in the two species is unlikely. Humans and pigs are under different selection pressures. Since naturally occurring SNPs differ in the two species, GWAS candidate loci in the two species may not significantly overlap when compared directly. The systems genetics and integrative network approach we used here is suitable for identifying potential causal genes underlying loci associated with complex human disease and, more importantly, to uncover potential biological processes underlying these causal loci.

For the rib number trait, we identified candidate genes in the Wnt and Notch pathways, which are known to influence rib development including *TGFB3* and *DAB2IP* as well as the known genes *MESP1* and *MESP2*. Using network approaches, we not only identified candidate genes, but also further dissected the mechanisms that mediate their effects on the phenotype. Abnormal rib number is associated with at least 19 different human diseases and syndromes (http://www.wrongdiagnosis.com/symptoms/abnormal_rib_number/view-all.htm), such as Herrmann-Opitz craniosynostosis, spondylocostal dysostosis, and Campomelia Cumming type. In humans, one extra rib in human can increase cancer risks 120-fold [41]. Our network analysis of the number of pig ribs not only revealed good markers for selecting pig breeds, but also showed that the pig is a good model for studying embryogenesis process in general and human congenital diseases.

An SNP in *NR6A1* has been implicated as the causal gene for vertebrae number at the chromosome 1 locus [45]. We genotyped this SNP and compared the genotypes of other SNPs in the chromosome 1 region. The genotypes of the *NR6A1* SNP and those around *NR6A1* on the 60 K chip were similar (Additional file 5: Figure S1c). Specifically, the genotypes of the *NR6A1* SNP and SNP marker DRGA0002465 (the center of the chromosome 1 locus in our study) were 90% identical. In addition, the *NR6A1* SNP and the neighboring SNPs on the 60 K SNP array had similar association results, and the *NR6A1* SNP explained less variation in rib number than DRGA0002465 (Additional file 5: Figure S1d), for which *DAB2IP* was implicated as the candidate causal gene in our study. These results suggest that *DAB2IP* is also likely to be a causal gene for rib number.

In addition to the three traits analyzed in the systems genetics study, there are over 40 phenotypic traits related to growth, fatness [60], meat quality [61], blood lipid profiles [62], and body strength [63] in the swine F2 cross. The three complex traits (anemia, leg length, and rib number) are not well studied in other animal models and are highly relevant to complex human diseases. By applying systems genetics and integrative network approaches, we were able to identify putative causal genes and mechanisms underlying the SNPs associated with these traits. However, systems biology approaches have some limitations in identifying causal genes of phenotypic traits. If gene expression data from relevant tissues is unavailable, one must rely on published networks and PPI networks, which have not been linked to many novel genes. In this case, the ability to find truly novel causal genes is limited. Alternative approaches such as genome-wide RNAi screening are needed to complement the systems biology approach.

## Conclusion

In summary, our results show that humans and pigs share similar genetic architecture for complex traits, providing proof of concept for using swine as a model organism for complex human diseases. Furthermore, the coherent genetic and phenotypic data generated in the study can provide a rich resource for future agriculture and human disease studies.

## Methods

### Swine F2 animals and phenotype recording

A large-scale F2 resource population was constructed by crossing two divergent pig breeds, White Duroc and Chinese Erhualian, as described previously [10]. Briefly, two White Duroc boars from Sygen PIC China Company and 17 Erhualian sows from three pig breeding farms were crossed as founder animals to produce F1 animals, and 59 F1 sows were randomly mated with 9 F1 boars to produce 1,912 F2 individuals from 110 full-sib families. All F2 pigs were kept under standard indoor conditions with natural lighting, fed formula feed three times a day, and given *ad libitum* access to water from nipple drinkers. At 46 days of age, all F2 piglets were weaned and moved to a nursery (males were castrated) until 120 days of age. Piglets were then transferred to a performance test station, where growth and feeding traits were recorded with an ACEMA 64 electronic auto-feed intake recording station, which phenotypes feeding behavior and food intake circadianly (ACEMO, Pontivy Cedex, France). At a mean age of 240 ± 3 days, the pigs were sent to a commercial slaughter facility and processed according to Chinese industry standards. All animal work was conducted in accordance with the guidelines for the care and use of experimental animals established by the Ministry of Agriculture of China.

Phenotypic values of growth, fertility, carcass, meat quality, immune capacity, behavior and morphological traits were recorded in this F2 pig populations. At the time of slaughter, blood samples were collected from the major artery vessels near the heart and morphological traits (including the number of ribs and the limb bone length) and blood biochemical traits were measured. Rib number was counted from the carcass. Both forelimb and hind limb were removed from the right side carcass of F2 animals and dissected from the limb. A large caliper was used to measure the length of five limb bones: humerus (total length from the head to the trochlea), scapula (the maximum straight line distance from the cavitas glenoidalis to the border of scapular cartilage), femur (total length from the greater trochanter to the intercondyloid fossa), ulna (length from the olecranon process to the styloid process) and tibia (length from the intercondylar eminence to the medial malleolus).

### SNP genotyping

502 F2 animals were successfully genotyped with a 60 K pig SNP chip (Illumina) [11] and an internally developed SNP set. The position of each SNP in the pig genome (Sscrofa10.2) was remapped with SOAP2 software. Quality control of genotypes was performed with the GenABEL procedure in R. SNPs with call rates < 95% or minor allele frequency < 15%, or Hardy Weinberg equilibrium (HWE) p-value were <5 × 10^−6^) and X-linked SNPs likely to be autosomal (odds > 1000) were excluded from further analysis. The associations between phenotypes and genome-wide SNP genotype data were analyzed with the single-marker association model in the GenABEL package [12] based on a mixed linear model implemented with the *mmscore* function of GenABEL in R package. Sex and batch were considered as fixed effects, and genetic co-variances among samples were considered by fitting a kinship matrix derived from genotypes of whole-genome SNP markers as *y* ∼ *sex* + *batch* + *g* + *G* , where *y* is a trait, *G* is kinship estimated based on genome-wide SNP data, and *g* is the genotype at a query SNP marker.

### Human liver transcriptional network

The human liver transcriptional network is the union of molecular Bayesian causal networks constructed from two human genetic gene expression studies [8,64]. In one study [8], liver samples of 466 Caucasian subjects were transcriptional profiled, 427 samples were genotyped and used in genetic gene expression analysis and Bayesian network reconstruction. Then, 8,188 genes were selected for inclusion in the network reconstruction process on the basis of two criteria: (1) variance of gene expression in the top 20% of gene expression variance; or (2) LOD scores of eQTLs of the genes were of genome-wide significance. Similarly, in the other study [64], liver tissues as well as omental adipose, subcutaneous adipose, and stomach tissues were collected from 1,008 patients during RYGB (Roux-en-Y gastric bypass); 651 liver samples were gene expression profiled and genotyped, and 7,593 genes with top gene expression variance were selected for Bayesian network reconstruction.

The selected genes were input into a Bayesian network reconstruction software package, RIMBANet-Reconstructing Integrative Molecular Bayesian Network [65], based on a previously described algorithm [65-68]. Genetics information was used to construct structure priors as follows: genes with cis eQTLs were allowed to be parent nodes of genes with coincident trans eQTLs, *p*(*cis* − > *trans*) = 1, but genes with trans eQTLs were not allowed to be parents of genes with cis eQTLs, *p*(*trans* − > *cis*) = 0. Bayesian Information Criterion (BIC) was used in the reconstruction process. One thousand Bayesian networks were reconstructed using different random seeds to start the reconstruction process. From the resulting set of 1000 networks generated by this process, edges that appeared in more than 30% of the networks were used to define a consensus network. In this consensus network, an edge was removed if 1) it was involved in a loop, and 2) it was the most weakly supported of all edges making up the loop.

The final human liver transcription network used in the anemia trait analysis is the union of the two Bayesian networks constructed based on the two data sets, which consists of 12,875 genes and 64,253 connections.

### Representation of transcription network of bone tissue

No large coherent set of bone expression data was available for building a human bone transcription network, so we used networks of tissues that are molecularly similar to bone. Adipocytes, osteoblasts, and chondrocytes share common progenitors and are close in lineage [69], and adipose and bone tissues share similar gene expression profiles based on the transcriptome body atlas [70]. Therefore, we used a network constructed from the adipose tissue to represent the bone network. A human gene regulatory network for omental fat was constructed based on 848 omental fat samples collected in the Greenawalt et al. study [64] from 1,008 patients at the time of RYGB as described above. These omental fat samples were gene expression profiled and genotyped. Then, 7,671 genes with large gene expression variance across the cohort were selected and input into Bayesian network reconstruction package RIMBANet as described above. The constructed omental fat network consisted of 13,979 connections and was used for representing a bone network in our network analysis.

### Human protein-protein interaction (PPI) network

The human PPI network was constructed by integrating human PPIs from several molecular interaction databases, both public (BIND, BioGRID, HPRD, MINT, Reactome, DIP, and IntAct) and commercial (Ingenuity, Proteome, MetaBase, and NetPro). Identifiers for the interacting genes identified in these databases were mapped to Entrez Gene IDs to obtain a unified naming system. Both directed regulations (e.g., “activates”, “inhibits” ) and undirected interactions (e.g., “binds”, “covalent binding”, “ppi”) in these databases were mapped to undirected edges in our PPI network, which consisted of 19,800 nodes and 65,056 interactions.

### Extracting a subnetwork from the Bayesian Networks and PPI network

To construct a subnetwork for a set of genes, we used genes in the input set as seeds and selected d-step neighbors of seeds (the shortest distance between a seed S and a neighbor node N is equal or less than d). When fewer than 50 seeds were used, d was set as 2; when more than 50 seeds were used, d was set as 1. Seeds and their d-step neighbors of seeds were nodes in the subnetwork, and links among nodes in the subnetworks were the same as in the whole Bayesian network.

### Collecting human GWAS candidates for MCV/MCH

Human GWAS candidates for traits mean corpuscular hemoglobin (MCH) / mean corpuscular volume (MCV) were retrieved from the NHGRI human GWAS catalog (http://www.genome.gov/gwastudies/), which covers 3 studies [17-19] with SNP association at genome-wide significance p-value cutoff of 1×10^−8^. Twenty-two unique loci and genes mapped to these loci were retrieved and listed in Additional file 2: Table S2.

### Statistical analyses of co-regulation in a network and functional enrichment

To measure closeness or potential of co-regulation of two genes in a network, we used the shortest distance between the two genes. Given a set of gene, the average shortest distance of all possible pairs was calculated. To assess significance of the observed average shortest distance, we randomly selected the same number of genes in the network and calculated their average shortest distance, and repeated the procedure 10,000 times. The probability of the observed average shortest distance expected by change was the number of the average shortest distance from randomly selected sets less than the observed shortest distance then divided by 10,000.

To identify potential functions of selected gene sets, we compared these gene sets with each GO biological process (GOBP) [71] and computed functional enrichment using Fisher’s Exact test (FET) and a conservative modification of FET as EASE scores [20]. Total 1352 GOBPs that consist of more than 10 genes and less than 1500 genes were tested, and only GOBPs with enrichment FET p-value <0.05/1352 were reported. To assess significance of functional enrichment of a subnetwork extracted from a network, we further adjusted enrichment p-values based on an empirical p-value distribution. Given a subnetwork in a whole network, we randomly shuffled node labels of the network, and then tested the overlap between the subnetwork and a biological process. The procedure was repeated 10,000 times. The probability of the observed p-value expected by chance is the number of the p-values from randomly shuffled networks less than the observed one divided by 10,000.

## Additional files

Additional file 1: Table S1. Loci significantly associated with MCH (mean corpuscular hemoglobin) in the swine F2 cross at false discover rate (FDR) <5% (corresponding *P* = 4.85 × 10^− 6^). Additional file 2: Table S2. Human GWAS candidates for MCH (mean corpuscular hemoglobin)/MCV (mean corpuscular volume) retrieved from the NHGRI GWAS catalog (www.genome.gov/gwastudies). All SNP associations pass the genomewide p-value cutoff 1e-8. Additional file 3: Table S3. Loci significantly associated with leg length in the swine F2 cross at false discover rate (FDR) <5% (corresponding *P* = 4.85 × 10^− 6^). Additional file 4: Table S4. Loci significantly associated with rib number in the swine F2 cross at false discover rate (FDR) <5% (corresponding *P* = *b*4.85 × 10^− 6^). Additional file 5: Figure S1. The genome-wide association result for the number of ribs. a) The global view of the association result shows that there are two significant loci on chromosomes 1 and 7 for the number of ribs. b) A zoom-in view of the chromosome 7 peak shows that there are two neighboring SNPs at the peak. c) The genotype of the SNP DRGA0002465 at the chromosome 1 peak is similar to the genotype of the *NR6A1* SNP. d) The zoom-in view of chromosome 1 peak shows that the SNP DRGA0002465 is more significantly associated with the number of ribs than the *NR6A1* SNP.

## References

[1] Wernersson R, Schierup MH, Jorgensen FG, Gorodkin J, Panitz F, Staerfeldt HH (2005). Pigs in sequence space: a 0.66X coverage pig genome survey based on shotgun sequencing. BMC Genomics.

[2] Grunwald KA, Schueler K, Uelmen PJ, Lipton BA, Kaiser M, Buhman K (1999). Identification of a novel Arg-Cys mutation in the LDL receptor that contributes to spontaneous hypercholesterolemia in pigs. J Lipid Res.

[3] Egidy G, Jule S, Bosse P, Bernex F, Geffrotin C, Vincent-Naulleau S (2008). Transcription analysis in the MeLiM swine model identifies RACK1 as a potential marker of malignancy for human melanocytic proliferation. Mol Cancer.

[4] Chen Y, Zhu J, Lum PY, Yang X, Pinto S, MacNeil DJ (2008). Variations in DNA elucidate molecular networks that cause disease. Nature.

[5] Emilsson V, Thorleifsson G, Zhang B, Leonardson AS, Zink F, Zhu J (2008). Genetics of gene expression and its effect on disease. Nature.

[6] Cerami E, Demir E, Schultz N, Taylor BS, Sander C (2010). Automated network analysis identifies core pathways in glioblastoma. PLoS One.

[7] Carro MS, Lim WK, Alvarez MJ, Bollo RJ, Zhao X, Snyder EY (2010). The transcriptional network for mesenchymal transformation of brain tumours. Nature.

[8] Schadt EE, Molony C, Chudin E, Hao K, Yang X, Lum PY (2008). Mapping the genetic architecture of gene expression in human liver. PLoS Biol.

[9] Musunuru K, Strong A, Frank-Kamenetsky M, Lee NE, Ahfeldt T, Sachs KV (2010). From noncoding variant to phenotype via SORT1 at the 1p13 cholesterol locus. Nature.

[10] Ren J, Guo YM, Ma JW, Huang LS (2006). Growth and meat quality QTL in pigs with special reference to a very large Erhualian × White Duroc resource population. 8th World Congress on Genetics Applied in Livestock Production: 2006; Belo Horizonte, MG, Brazil.

[11] Ramos AM, Crooijmans RP, Affara NA, Amaral AJ, Archibald AL, Beever JE (2009). Design of a high density SNP genotyping assay in the pig using SNPs identified and characterized by next generation sequencing technology. PLoS One.

[12] Aulchenko YS, Ripke S, Isaacs A, van Duijn CM (2007). GenABEL: an R library for genome-wide association analysis. Bioinformatics.

[13] Johansson A, Pielberg G, Andersson L, Edfors-Lilja I (2005). Polymorphism at the porcine Dominant white/KIT locus influence coat colour and peripheral blood cell measures. Anim Genet.

[14] Zou Z, Ren J, Yan X, Huang X, Yang S, Zhang Z (2008). Quantitative trait loci for porcine baseline erythroid traits at three growth ages in a White Duroc x Erhualian F(2) resource population. Mamm Genome.

[15] Pielberg G, Olsson C, Syvanen AC, Andersson L (2002). Unexpectedly high allelic diversity at the KIT locus causing dominant white color in the domestic pig. Genetics.

[16] Recalcati S, Alberghini A, Campanella A, Gianelli U, De Camilli E, Conte D (2006). Iron regulatory proteins 1 and 2 in human monocytes, macrophages and duodenum: expression and regulation in hereditary hemochromatosis and iron deficiency. Haematologica.

[17] Li J, Glessner JT, Zhang H, Hou C, Wei Z, Bradfield JP (2013). GWAS of blood cell traits identifies novel associated loci and epistatic interactions in Caucasian and African-American children. Hum Mol Genet.

[18] Kamatani Y, Matsuda K, Okada Y, Kubo M, Hosono N, Daigo Y (2010). Genome-wide association study of hematological and biochemical traits in a Japanese population. Nat Genet.

[19] Ganesh SK, Zakai NA, van Rooij FJ, Soranzo N, Smith AV, Nalls MA (2009). Multiple loci influence erythrocyte phenotypes in the CHARGE Consortium. Nat Genet.

[20] Hosack DA, Dennis G, Sherman BT, Lane HC, Lempicki RA (2003). Identifying biological themes within lists of genes with EASE. Genome Biol.

[21] Fillios LC, Andrus SB, Naito C (1961). Coronary lipid deposition during chronic anemia or high altitude exposure. J Appl Physiol.

[22] Fujii T, Shimizu H (1973). Investigations on serum lipid components and serum vitamin E in iron deficiency anemia. J Nutr Sci Vitaminol.

[23] Ohira Y, Edgerton VR, Gardner GW, Senewiratne B (1980). Serum lipid levels in iron deficiency anemia and effects of various treatments. J Nutr Sci Vitaminol.

[24] Ozsoylu S (1994). Lipid peroxidation in iron deficiency anemia. Acta Haematol.

[25] Pesillo SA, Freeman LM, Rush JE (2004). Assessment of lipid peroxidation and serum vitamin E concentration in dogs with immune-mediated hemolytic anemia. Am J Vet Res.

[26] Ahamed M, Kumar A, Siddiqui MK (2006). Lipid peroxidation and antioxidant status in the blood of children with aplastic anemia. Clin Chim Acta.

[27] Ozdemir A, Sevinc C, Selamet U, Turkmen F (2007). The relationship between iron deficiency anemia and lipid metabolism in premenopausal women. Am J Med Sci.

[28] Oztas YE, Sabuncuoglu S, Unal S, Ozgunes H, Ozgunes N (2011). Hypocholesterolemia is associated negatively with hemolysate lipid peroxidation in sickle cell anemia patients. Clin Exp Med.

[29] McClung JP, Karl JP (2009). Iron deficiency and obesity: the contribution of inflammation and diminished iron absorption. Nutr Rev.

[30] Ganz T (2003). Hepcidin, a key regulator of iron metabolism and mediator of anemia of inflammation. Blood.

[31] del Giudice EM, Santoro N, Amato A, Brienza C, Calabro P, Wiegerinck ET (2009). Hepcidin in obese children as a potential mediator of the association between obesity and iron deficiency. J Clin Endocrinol Metab.

[32] Lango Allen H, Estrada K, Lettre G, Berndt SI, Weedon MN, Rivadeneira F (2010). Hundreds of variants clustered in genomic loci and biological pathways affect human height. Nature.

[33] Mao H, Guo Y, Yang G, Yang B, Ren J, Liu S (2008). A genome-wide scan for quantitative trait loci affecting limb bone lengths and areal bone mineral density of the distal femur in a White Duroc x Erhualian F2 population. BMC Genet.

[34] Soranzo N, Rivadeneira F, Chinappen-Horsley U, Malkina I, Richards JB, Hammond N (2009). Meta-analysis of genome-wide scans for human adult stature identifies novel Loci and associations with measures of skeletal frame size. PLoS Genet.

[35] Gudbjartsson DF, Walters GB, Thorleifsson G, Stefansson H, Halldorsson BV, Zusmanovich P (2008). Many sequence variants affecting diversity of adult human height. Nat Genet.

[36] Reyes M, Lund T, Lenvik T, Aguiar D, Koodie L, Verfaillie CM (2001). Purification and ex vivo expansion of postnatal human marrow mesodermal progenitor cells. Blood.

[37] Pignolo RJ, Xu M, Russell E, Richardson A, Kaplan J, Billings PC (2011). Heterozygous inactivation of Gnas in adipose-derived mesenchymal progenitor cells enhances osteoblast differentiation and promotes heterotopic ossification. J Bone Miner Res.

[38] Hensen K, Braem C, Declercq J, Van Dyck F, Dewerchin M, Fiette L (2004). Targeted disruption of the murine Plag1 proto-oncogene causes growth retardation and reduced fertility. Dev Growth Differ.

[39] Karim L, Takeda H, Lin L, Druet T, Arias JA, Baurain D (2011). Variants modulating the expression of a chromosome domain encompassing PLAG1 influence bovine stature. Nat Genet.

[40] King J, Roberts R (1960). Carcass length in the bacon pig; Its association with vertebrae numbers and prediction from radiographs of the young pig. Animal Prod.

[41] Galis F, Metz JA (2003). Anti-cancer selection as a source of developmental and evolutionary constraints. Bioessays.

[42] Edwards DB, Ernst CW, Raney NE, Doumit ME, Hoge MD, Bates RO (2008). Quantitative trait locus mapping in an F2 Duroc x Pietrain resource population: II. Carcass Meat Qual Traits J Anim Sci.

[43] Zhang J, Xiong Y, Zuo B, Lei M, Jiang S, Li F (2007). Detection of quantitative trait loci associated with several internal organ traits and teat number trait in a pig population. J Genet Genomics.

[44] Harmegnies N, Davin F, De Smet S, Buys N, Georges M, Coppieters W (2006). Results of a whole-genome quantitative trait locus scan for growth, carcass composition and meat quality in a porcine four-way cross. Anim Genet.

[45] Mikawa S, Morozumi T, Shimanuki S, Hayashi T, Uenishi H, Domukai M (2007). Fine mapping of a swine quantitative trait locus for number of vertebrae and analysis of an orphan nuclear receptor, germ cell nuclear factor (NR6A1). Genome Res.

[46] Mikawa S, Sato S, Nii M, Morozumi T, Yoshioka G, Imaeda N (2011). Identification of a second gene associated with variation in vertebral number in domestic pigs. BMC Genet.

[47] Gibb S, Maroto M, Dale JK (2010). The segmentation clock mechanism moves up a notch. Trends Cell Biol.

[48] Dunker N, Krieglstein K (2002). Tgfbeta2 −/− Tgfbeta3 −/− double knockout mice display severe midline fusion defects and early embryonic lethality. Anat Embryol.

[49] Yang LT, Kaartinen V (2007). Tgfb1 expressed in the Tgfb3 locus partially rescues the cleft palate phenotype of Tgfb3 null mutants. Dev Biol.

[50] Wang Z, Tseng CP, Pong RC, Chen H, McConnell JD, Navone N (2002). The mechanism of growth-inhibitory effect of DOC-2/DAB2 in prostate cancer. Characterization of a novel GTPase-activating protein associated with N-terminal domain of DOC-2/DAB2. J Biol Chem.

[51] Huang CL, Cheng JC, Kitajima K, Nakano T, Yeh CF, Chong KY (2010). Disabled-2 is required for mesoderm differentiation of murine embryonic stem cells. J Cell Physiol.

[52] Jiang Y, Luo W, Howe PH (2009). Dab2 stabilizes Axin and attenuates Wnt/beta-catenin signaling by preventing protein phosphatase 1 (PP1)-Axin interactions. Oncogene.

[53] Aulehla A, Wiegraebe W, Baubet V, Wahl MB, Deng C, Taketo M (2008). A beta-catenin gradient links the clock and wavefront systems in mouse embryo segmentation. Nat Cell Biol.

[54] Chaudhury A, Hussey GS, Ray PS, Jin G, Fox PL, Howe PH (2010). TGF-beta-mediated phosphorylation of hnRNP E1 induces EMT via transcript-selective translational induction of Dab2 and ILEI. Nat Cell Biol.

[55] Takahashi Y, Yasuhiko Y, Kitajima S, Kanno J, Saga Y (2007). Appropriate suppression of Notch signaling by Mesp factors is essential for stripe pattern formation leading to segment boundary formation. Dev Biol.

[56] Sasaki N, Kiso M, Kitagawa M, Saga Y (2011). The repression of Notch signaling occurs via the destabilization of mastermind-like 1 by Mesp2 and is essential for somitogenesis. Development.

[57] Groenen MA, Archibald AL, Uenishi H, Tuggle CK, Takeuchi Y, Rothschild MF (2012). Analyses of pig genomes provide insight into porcine demography and evolution. Nature.

[58] Kelada SN, Aylor DL, Peck BC, Ryan JF, Tavarez U, Buus RJ (2012). Genetic analysis of hematological parameters in incipient lines of the collaborative cross. G3 (Bethesda).

[59] Davis RC, van Nas A, Bennett B, Orozco L, Pan C, Rau CD (2013). Genome-wide association mapping of blood cell traits in mice. Mamm Genome.

[60] Ai H, Ren J, Zhang Z, Ma J, Guo Y, Yang B (2012). Detection of quantitative trait loci for growth- and fatness-related traits in a large-scale White Duroc x Erhualian intercross pig population. Anim Genet.

[61] Ma J, Yang J, Zhou L, Zhang Z, Ma H, Xie X (2013). Genome-wide association study of meat quality traits in a White DurocxErhualian F2 intercross and Chinese Sutai pigs. PLoS One.

[62] Chen C, Yang B, Zeng Z, Yang H, Liu C, Ren J (2013). Genetic dissection of blood lipid traits by integrating genome-wide association study and gene expression profiling in a porcine model. BMC Genomics.

[63] Guo YM, Zhang XF, Ren J, Ai HS, Ma JW, Huang LS (2013). A joint genomewide association analysis of pig leg weakness and its related traits in an F2 population and a Sutai population. J Anim Sci.

[64] Greenawalt DM, Dobrin R, Chudin E, Hatoum IJ, Suver C, Beaulaurier J (2011). A survey of the genetics of stomach, liver, and adipose gene expression from a morbidly obese cohort. Genome Res.

[65] Zhu J, Sova P, Xu Q, Dombek KM, Xu EY, Vu H (2012). Stitching together multiple data dimensions reveals interacting metabolomic and transcriptomic networks that modulate cell regulation. PLoS Biol.

[66] Zhu J, Lum PY, Lamb J, GuhaThakurta D, Edwards SW, Thieringer R (2004). An integrative genomics approach to the reconstruction of gene networks in segregating populations. Cytogenet Genome Res.

[67] Zhu J, Wiener MC, Zhang C, Fridman A, Minch E, Lum PY (2007). Increasing the Power to Detect Causal Associations by Combining Genotypic and Expression Data in Segregating Populations. PLoS Comput Biol.

[68] Zhu J, Zhang B, Smith EN, Drees B, Brem RB, Kruglyak L (2008). Integrating large-scale functional genomic data to dissect the complexity of yeast regulatory networks. Nat Genet.

[69] Nuttall ME, Patton AJ, Olivera DL, Nadeau DP, Gowen M (1998). Human trabecular bone cells are able to express both osteoblastic and adipocytic phenotype: implications for osteopenic disorders. J Bone Miner Res.

[70] Bono H, Yagi K, Kasukawa T, Nikaido I, Tominaga N, Miki R (2003). Systematic expression profiling of the mouse transcriptome using RIKEN cDNA microarrays. Genome Res.

[71] Ashburner M, Ball CA, Blake JA, Botstein D, Butler H, Cherry JM (2000). Gene ontology: tool for the unification of biology. Gene Ontol Consortium Nat Genet.

