# Supplementary material for: A systems genetics study of swine illustrates mechanisms underlying human phenotypic traits
Source: BMC Genomics. 2015 Feb 14;16(1):88. doi: 10.1186/s12864-015-1240-y (PMC4336704; doi:10.1186/s12864-015-1240-y)
Supplement: Additional file 5: Figure S1. — The genome-wide association result for the number of ribs. a) The global view of the association result shows that there are two significant loci on chromosomes 1 and 7 for the number of ribs. b) A zoom-in view of the chromosome 7 peak shows that there are two neighboring SNPs at the peak. c) The genotype of the SNP DRGA0002465 at the chromosome 1 peak is similar to the genotype of the NR6A1 SNP. d) The zoom-in view of chromosome 1 peak shows that the SNP DRGA0002465 is more significantly associated with the number of ribs than the NR6A1 SNP. [file 12864_2015_1240_MOESM5_ESM.docx]

**Additional file 5: Figure S1**

**Supplementary Information for**

**A Systems Genetics Study of Swine Illustrates Mechanisms Underlying Human Phenotypic Traits**

Jun Zhu^1,2#†^, Congying Chen^1#^, Bin Yang^1^, Yuanmei Guo^1^, Huashui Ai^1^, Jun Ren^1^, Zhiyu Peng^3^, Zhidong Tu^2^, Xia Yang^4^, Qingying Meng^4^, Stephen Friend^5^, Lusheng Huang^1,†^

^1^Jiangxi Agricultural University, Nanchang, Jiangxi, China; ^2^Icahn Institute for Genomics and Multiscale Biology, Icahn School of Medicine at Mount Sinai, New York, NY, USA; ^3^BGI, Shenzhen, Guangdong, China; ^4^Department of Integrative Biology and Physiology, University of California at Los Angeles, Los Angeles, CA, USA; ^5^Sage Bionetworks, Seattle, WA, USA;

Jun Zhu ([jun.zhu@mssm.edu](mailto:jun.zhu@mssm.edu))

Congying Chen([chcy75@hotmail.com](mailto:chcy75@hotmail.com))

Bin Yang ([ybb_wx@hotmail.com](mailto:ybb_wx@hotmail.com))

Yuanmei Guo ([gyuanmei@hotmail.com](mailto:gyuanmei@hotmail.com))

Huashui Ai ([aihsh@hotmail.com](mailto:aihsh@hotmail.com))

Jun Ren ([renjunjxau@hotmail.com](mailto:renjunjxau@hotmail.com))

Zhiyu Peng ([pengbgi@gmail.com](mailto:pengbgi@gmail.com))

Zhidong Tu ([zhidong.tu@mssm.edu](mailto:zhidong.tu@mssm.edu))

Xia Yang ([xyang123@ucla.edu](mailto:xyang123@ucla.edu))

Qingying Meng ([qingyingmeng@ucla.edu](mailto:qingyingmeng@ucla.edu))

Stephen Friend ([friend@sagebase.org](mailto:friend@sagebase.org))

Lusheng Huang ([Lushenghuang@hotmail.com](mailto:Lushenghuang@hotmail.com))

# co-first authors

†Correspondence should be addressed to

Dr. Jun Zhu

Professor, Genetics and Genomic Sciences

Icahn Institute of Genomics and Multiscale Biology

Icahn School of Medicine at Mount Sinai

New York, NY 10029

Tel: 212-659-8942

Email: [jun.zhu@mssm.edu](mailto:jun.zhu@mssm.edu)

Dr. Lusheng Huang

Professor, President of Jiangxi Agricultural University

Nanchang, Jiangxi, China

Tel: 0086-791-8380-5967

Email: [lushenghuang@hotmail.com](mailto:lushenghuang@hotmail.com)

**Supplementary Results**

**Integrative network analysis of swine and human GWAS candidate genes for MCH**

Bone marrow, kidney, and liver are important tissues for red blood cell production and homeostasis. With a human liver transcriptional network available (see Materials and Methods for details) we examined how these genes are regulated in the network. There are 12,875 genes in the human liver network. 7 of 16 swine GWAS candidates for MCH and 28 of 47 human GWAS candidates were included the network. These candidate genes were likely to be transcriptionally co-regulated in human liver. Subnetworks around swine candidates for MCH contained 276 genes, and were significantly enriched in the GO biological process cellular lipid metabolism, which consists of 505 genes in the network (38 genes in the overlap, fold enrichment =3.5, FET p-value=$1.2\times10^{-11}$, “p=phyper(37,276,12599,505,lower.tail=FALSE,log.p=FALSE)” in R or “p=1-hygecdf(38-1, 12875, 276, 505)” in Matlab, EASE score =$4.5\times10^{-11}$ “p=phyper(37-1,276-1,12600,505,lower.tail=FALSE,log.p=FALSE)” in R or “p=1-hygecdf(38-2, 12875, 276-1, 505)” in Matlab). Similarly, subnetworks around human GWAS candidates consisted of 1568 genes, and were enriched in the GO biological processes immune response (consisting of 853 genes in the network) and lipid metabolism (consisting of 594 genes in the network) (185 and 112 genes in the overlaps, fold enrichments = 1.78 and 1.55, FETest p-values= $3.3\times10^{-16}$ and $1.1\times10^{-6}$, EASE scores = $7.8\times10^{-16}$and $1.8\times10^{-6}$, respectively) . The combined subnetworks around swine and human GWAS candidate genes consisted of 1776 genes, which were enriched for the GO biological process cellular lipid metabolism (consisting of 505 genes in the network) (126 in the overlap, fold enrichment=1.8, Fisher’s Exact Test p-value = $6.3\times10^{-12}$, EASE score = $1.3\times10^{-11}$) .

**Supplementary Figures**

**Additional file 5: Figure S1** The genome-wide association result for the number of ribs. a) The global view of the association result shows that there are two significant loci on chromosomes 1 and 7 for the number of ribs. b) A zoom-in view of the chromosome 7 peak shows that there are two neighboring SNPs at the peak. c) The genotype of the SNP DRGA0002465 at the chromosome 1 peak is similar to the genotype of the *NR6A1* SNP. d) The zoom-in view of chromosome 1 peak shows that the SNP DRGA0002465 is more significantly associated with the number of ribs than the *NR6A1* SNP.


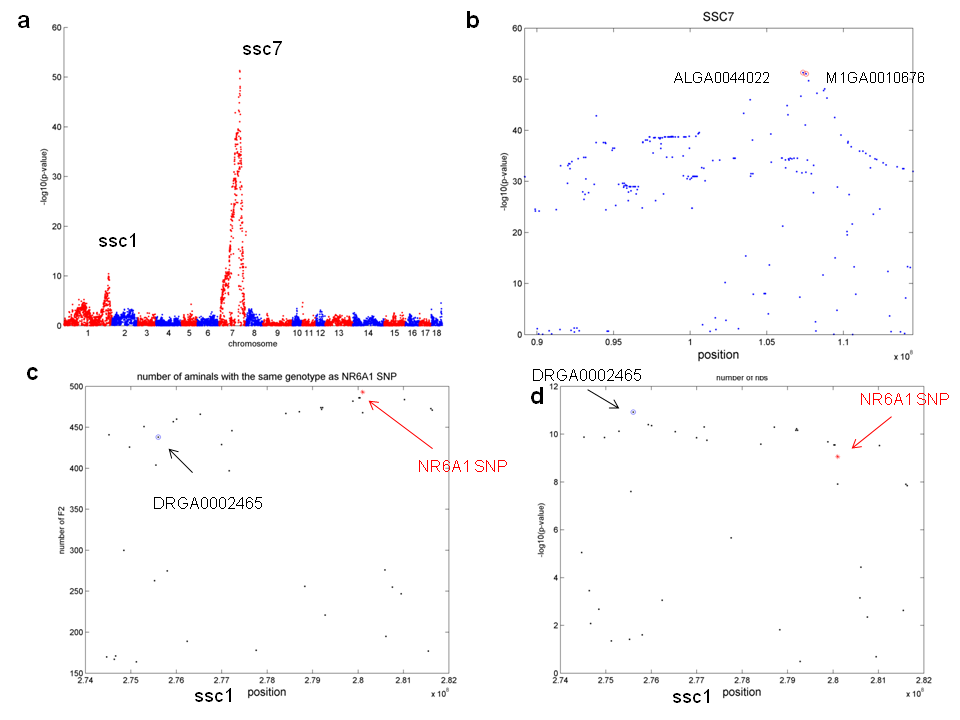


**Supplementary Tables**

**Additional file 1: Table S1** Loci significantly associated with MCH (mean corpuscular hemoglobin) in the swine F2 cross at false discover rate (FDR) <5% (corresponding *P*=$4.85\times10^{-6}$).

**Additional file 2: Table S2** Human GWAS candidates for MCH (mean corpuscular hemoglobin)/MCV (mean corpuscular volume) retrieved from the NHGRI GWAS catalog (www.genome.gov/gwastudies). All SNP associations pass the genomewide p-value cutoff 1e-8.

**Additional file 3: Table S3** Loci significantly associated with leg length in the swine F2 cross at false discover rate (FDR) <5% (corresponding *P*=$4.85\times10^{-6}$).

**Additional file 4: Table S4** Loci significantly associated with rib number in the swine F2 cross at false discover rate (FDR) <5% (corresponding *P*=$4.85\times10^{-6}$).
